# Effects of Multiple Filters on Liver Tumor Segmentation From CT Images

**DOI:** 10.3389/fonc.2021.697178

**Published:** 2021-10-01

**Authors:** Vi Thi-Tuong Vo, Hyung-Jeong Yang, Guee-Sang Lee, Sae-Ryung Kang, Soo-Hyung Kim

**Affiliations:** ^1^ Department of Artificial Intelligence Convergence, Chonnam National University, Gwangju, South Korea; ^2^ Department of Nuclear Medicine, Chonnam National University Hwasun Hospital, Gwangju, South Korea

**Keywords:** liver tumor, CT image, segmentation, multiple filter, convolutional neural network

## Abstract

Segmentation of liver tumors from Computerized Tomography (CT) images remains a challenge due to the natural variation in tumor shape and structure as well as the noise in CT images. A key assumption is that the performance of liver tumor segmentation depends on the characteristics of multiple features extracted from multiple filters. In this paper, we design an enhanced approach based on a two-class (liver, tumor) convolutional neural network that discriminates tumor as well as liver from CT images. First, the contrast and intensity values in CT images are adjusted and high frequencies are removed using Hounsfield units (HU) filtering and standardization. Then, the liver tumor is segmented from entire images with multiple filter U-net (MFU-net). Finally, a quantitative analysis is carried out to evaluate the segmentation results using three different methods: boundary-distance-based metrics, size-based metrics, and overlap-based metrics. The proposed method is validated on CT images from the 3Dircadb and LiTS dataset. The results demonstrate that the multiple filters are useful for extracting local and global feature simultaneously, minimizing the boundary distance errors, and our approach demonstrates better performance in heterogeneous tumor regions of CT images.

## 1 Introduction

Computerized Tomography (CT) of the abdomen is a diagnostic imaging method that is often used in clinical practice and to advance research on liver diseases. Among the many liver-related diseases, hepatocellular carcinoma (HCC) is the most common primary liver cancer. HCC occurs most often in patients with chronic liver diseases, such as cirrhosis, hepatitis, and liver infection. HCC often appears as pale masses in the liver which may be unifocal, multifocal, or diffusely infiltrative at the time of detection. The manifestations of HCC can be divided into massive, infiltrative and nodular. Each has different radiological features. The massive type is characterized by a large tumor that occupies almost the entire right or left lobe with an ambiguous or irregular boundary. The diffuse/infiltrative type consists of multiple diffuse proliferative tumor nodules throughout the liver. The nodular type is characterized by a small HCC, up to around 2 cm in diameter, and can be divided into two subtypes: a distinctly nodular type and an indistinctly nodular type. The distinctly nodular type is represented by a clear nodule with a fibrous capsule and/or fibrous septa in about 50% of cases; these are already advanced tumors despite their small size (1). Meanwhile, an indistinctly nodular tumor appears only vaguely nodular, with unclear margins. However, most are detected as hypoechoic or hyperechoic nodular lesions on an ultrasound exam and can be interpreted as “carcinoma *in situ*” of the liver. Currently, indistinct nodular HCCs are the smallest type of HCC that can be clinically detected. The goals of evaluating a hepatic nodule on CT images in a patient with liver cirrhosis include not only identifying the nature of the lesion but also estimating the hepatic extension of the neoplasia and any possible localization in extrahepatic sites (2). From there, a clinician can propose a suitable treatment based on the exact staging of the disease. Identifying small HCC nodules in a cirrhotic liver with an irregular parenchymal pattern is not easy. The level of contrast between the liver and the surrounding area is low and complex. Liver tumors are varied and complicated in shape and position and often do not have clear edges. Contrast factors are commonly known as noise elements in CT images. Therefore, segmentation of liver tumors is considered a challenging task.

A clinically trained expert usually makes a liver tumor diagnosis on the basis of many years of experience by manually identifying liver ROIs on one or more CT slices. However, manual identification is resource and time intensive for clinical practitioners and cannot be scaled up for large-scale medical image data purposes. Therefore, development of an automatic liver tumor segmentation algorithm is essential.

**Table 1** presents a summary of the liver tumor segmentation methods. Thresholding is the first simple and effective method that was proposed to automatically separate tumors from liver and background tissue (3, 19). Then, spatial regularization methods were developed that extract tumor regions based on size, shape, surface or spatial information, known as morphologies (4). In addition, a fuzzy classification-based tool (6), AdaBoost, was built which trains an algorithm using textural features (10), and has become the most prominent supervised classification method. Clustering methods include fuzzy c-means clustering with segmentation refinement using deformable models (8) and Ek-means (7). Among deep learning methods, Han, the winner of the first round in the LiTS challenge, proposed the 2.5D DCNN model, which uses a series of contiguous slices as inputs and creates a segmentation map that corresponds to the center slice. The model has 32 layers and uses the long-range concatenation connections of U-Net (20) in conjunction with the short-range residual connections of ResNet (21) simultaneously. H-DenseUNet (18) is a combination of a 2D DenseUNet and a 3D counterpart. A 2D DenseUNet is used to efficiently extract the intra-slice features. The 3D counterpart is used to hierarchically combine the volumetric contexts according to an auto-context algorithm. A hybrid feature fusion (HFF) layer is then applied to join the intraslice feature with interslice features. H-DenseUNet is not an end-to-end model. However, this method achieves state-of-the-art tumor segmentation results and competitive liver segmentation performance.

**Table 1:** Liver tumor segmentation methods.

Author	Dataset		Methodology
	No. of sample	Description	
(3)	35	Private	Thresholding
(4)	10	MICCAI 2008	Adaptive thresholding and morphological processing
(5)	68	MICCAI 2008	Histogram analysis
(6)	–	MICCAI 2008	Level method with spiral-scanning, supervised fuzzy pixel classification
(7)	21	Private	K-mean
(8)	10	LTS08	Implicit surface evolution
(9)	27	Private	Texture-based Omni-directional deformable surface model
(10)	16	MICCAI2007	Adaboost
(11)			Scale-adaptive supervoxel-based random forest
(12)	78	MICCAI-Sliver07, 3Dircadb1	Convolutional neural network and graph cut
(13)	_	MICCAI 2008	Graph-cut and watershed
(14)	10	MICCAI 2008	Entropy based multi-Thresholding
(15)	10	MICCAI 2008	Iterative Bayesian
(16)	_	3Dircadb, JDRD	FCNs
(17)	201	LiTS	CDNN
(18)	201	LiTS	HdenseUnet

In semantic medical image processing fields, the U-net model is one of the most popular fully convolutional network models. The U-net architecture is a pixel-to-pixel fully convolutional network with a skip connection between the encoder path and the decoder path. Its greatest advantage comes from the combination of location information from the downsampling path and the contextual information from the upsampling path. This is necessary to produce a good segmentation prediction based on location and context, combining general information from all images. However, the standard U-Net architecture contains only a few layers and, therefore, is not currently deep enough to address outstanding issues in the medical field.

One of the most promising paths forward involves adding more layers directly to the network to make a deeper network. The concept of multiple layers was first introduced in (22) as the simplest inception model. The main advantage of the inception module is that it improves the utilization rate of computing resources by increasing the depth and width of the network while keeping the computational budget constant (23). Each filter is presented with specific features or patterns in the original image. The filter is shifted several times and then applied at different image positions until the general image has been detailed. In this way, training efficiency and accuracy are improved.

Inspired by the works mentioned above but unlike these current methods, in an effort to develop a deep learning network appropriate for medical image segmentation tasks, we proposed an architecture that combines the multiple filter module based on the U-Net architecture, named MFU-net. Our methods can adaptively use the features from the multiple filter convolution for diminishing the boundary distance errors. The details are as follows:

1. Analyzes the effectiveness of the mean value of each image in the contrast and gamma enhancement automatically.
2. Based on GoogLeNet, to make the network wider without causing gradients to vanish, every convolutional layer is replaced by a multiple filter block with nonuniquely sized convolutional kernels in each block.
3. Based on the architecture of U-Net, the encoder path and decoder path are used in a network with skip connections to transmit feature maps directly from the downsampling process to the upsampling process. The encoder path is constructed from Resnet18. The decoder path is proposed by combining multiple filter blocks together. This contributes to improve the segmentation performance in the boundary-distance-based metrics.

Our end-to-end learning can predict liver and tumor simultaneously. This not only gains the competitive performance of liver segmentation but also contributes to minimizing the boundary distance error between the predicted and labeled tumors, which are known to be small and varied in size and shape.

## 2 Materials and Methods

**Figure 1:**
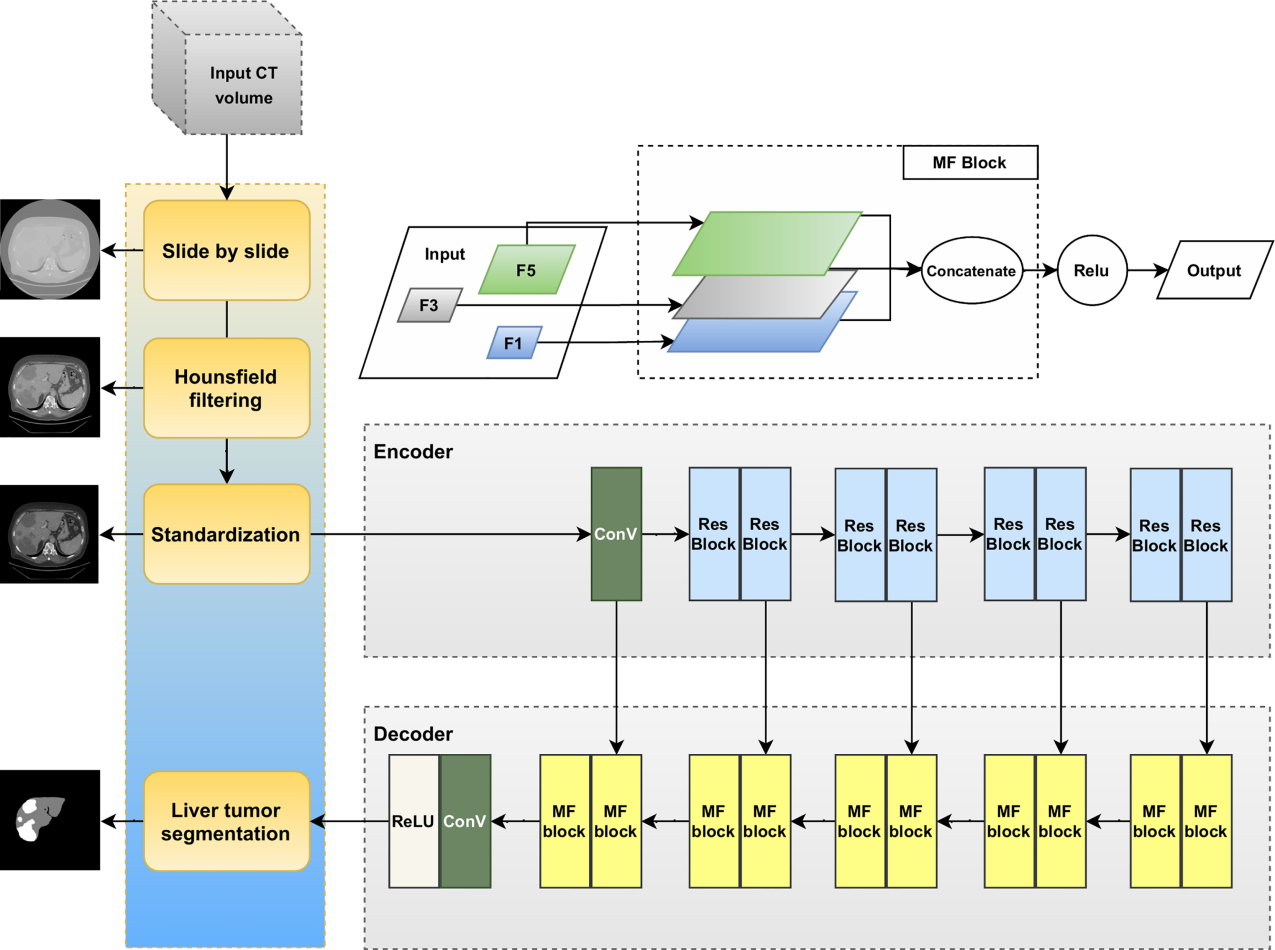
Proposed phases for automatic liver tumor segmentation.

This section provides an explanation of the method used to segment liver tumors in an end-to-end manner; a schematic illustration of the pipeline is presented in **Figure 1**. The liver tumor segmentation pipeline consists of two main sections: preprocessing (Hounsfield filtering, standardization) and liver tumor segmentation. The liver tumor segmentation was designed using a deep convolutional neural network described on the right-hand side including the Encoder and Decoder paths.

### 2.1 Preprocessing

Data preprocessing is the first important step before any deep learning model can be applied because machine learning and deep learning algorithms learn from the data, and the learning output depends on data to solve a particular problem (24, 25). The entire dataset needs to be normalized and outliers removed. The processing stage is accomplished through (1).


(1) $$I'=H_{I}+S_{\left(I,\mu ,\alpha ,\beta ,\gamma \right)},I,I'\in {\mathbb{R}}^{512\times 512}$$

Functions *H* and *S* are defined as (2) and (3).

With CT scans, the most common means of calculating some values relative to the liver is filtering using Hounsfield units (HUs). It helps to focus concentration on the important aspects of each segmentation task.

Therefore, with an input CT image, *I* ∈ℝ^512×512^, we denote function *H*, or Hounsfield, to remove the non-liver-related organs and tissues.


(2) $$H=slope\times I_{\left(x,y\right)}+Intercept$$

where slope = 0.00390625, Intercept = 0.1 × min(*I*) since min(*I*) < = 0 and Intercept = -min(*I*) since min(*I*) > 0.

**Figure 2:**
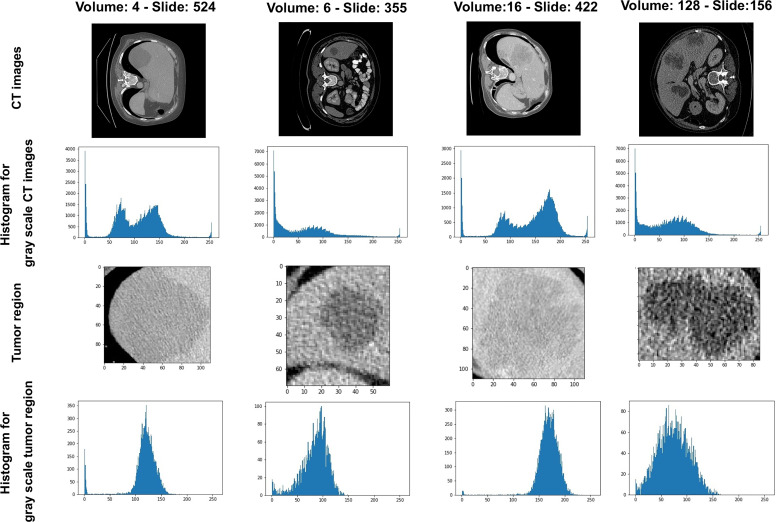
Example of CT images and tumor regions with their histogram of gray-scale images, respectively.

We recognize that there is extensive heterogeneity in liver and tumor contrast among slices. As shown in **Figure 2**, the contrast, brightness, size, and shape of the liver and liver tumor vary greatly among CT images. For more detail, **Figure 2** illustrates some examples of the histogram of CT images with corresponding tumor regions. The pixel value of 0 represents the background regions. After HU filtering, we can differentiate between cancerous and noncancerous areas. However, there are differences between samples in brightness, contrast, and saturation, leading to harder learning and higher errors. We also assumed that the mean value of an image influences the gamma and contrast balance in the whole dataset. As a result, we process the image for more balance and stay within a more synchronous range.

Therefore, the next step is standardization. Given an input, ∈ℝ^512×512^, we define the transform function, 𝒮, with some parameters, *α*, *β*, and *γ*. The threshold (selected through experimentation) was used to balance grammar and contrast among volumes.


(3) $${𝒮}_{I,\mu ,\alpha ,\beta ,\gamma }=(\alpha \cdot I_{\left(x,y\right)}+\beta )+{\left(\frac{I_{\left(x,y\right)}}{255}\right)}^{\gamma }\times 255$$

where *µ* is the mean of the image matrix. The values of *α* and *β* are used to scale the input. The value of *γ* is used to adjust the contrast or the brightness of the image. All of these parameters (*α*, *β*, and *γ*) are empirically set in our experiments excluding *µ*. In particular, the value of *α* in [1, 2], the value of *β* in [1, 1.2] and the value of *γ* in [1.5, 2] of each case. We used the mean value of the image as the threshold value for applying these *α*, *β*, and *γ* values corresponding with specialized cases.

### 2.2 Multiple Filter Blocks

This section introduces the multiple filter block (MF block). Because of the variation in tumor shape and location, the MF block applies a multi-filter size on the original layer instead of applying the same filter to one input layer. Our goal is to leverage the advantages of multiple filters with a filter hierarchy. Therefore, we take the outputs from the three convolutions with different filter sizes and concatenate them together to capture the features of each one. The result is nearly identical to the output of the Inception-like block described earlier.

The multifilter block is a stack of three convolution layers with different kernel size: 1 × 1, 3 × 3 and 5 × 5. The first convolution kernel has a filter size of 1 × 1. We apply this convolution to reduce the size of the input vector as well as extract local feature. While small kernels extract small complex features, the large kernel extract simpler features. Therefore, the next convolutional layer was set to 3 × 3 convolution kernel and uses a down-sampling size of 2 to obtain the global features. The last convolutional layer has a kernel size of 5 × 5 and a downsampling size of 2. The purpose of using this kernel size is to spread across the image and extract both liver and tumor features simultaneously.

Each filter learns different features. Therefore, the multi-filter block is used to deal with the issue by increasing the filter size instead of iteratively alleviating the image size. The combining multiple convolution layers is to yield a better result.

Multiple filter block can be performed as in (4).


(4) $$x'=R(x)=\max(0,x)=\max(0,F_{\left(x,f(1\times 1\right)}⊕F_{\left(x,f(3\times 3)\right)}⊕F_{\left(x,f(5\times 5)\right)})x\in {\mathbb{R}}^{n\times 512\times 512\times 1}$$

where ℛ is the Rectified Linear activation function or ReLU, ℱ is the convolutional layer, *f* is the filter with various size (1 × 1, 3 × 3, and 5 × 5), *x* is the input of multiple filter block (MF block), *x*' is the output of MF block, and ⊕ represents concatenation operation.

### 2.3 Proposed Liver Tumor Segmentation Method

Inspired by the attention U-Net model, the Inception module, we proposed a convolutional neural network that integrates multiple filters into the original U-Net. The proposed Multiple Filter U-Net architecture, denoted MFU-Net, is demonstrated in the right side of **Figure 1**. This model contains the encoder path and the decoder path. The encoder path likes Restnet18. The decoder path consists of 10 multiple filter blocks. Between two paths, a single skip connection is deployed.

## 3 Results

### 3.1 Evaluation Metrics

The evaluation metrics for segmentation are inconsistent, and they tend to be sensitive to one or more different types of segmentation errors such as size, position, and shape of an object (26). None of the metrics can cover all of these types of errors. Therefore, we evaluated the liver tumor segmentation quality of our algorithm based on boundary-distance-based metrics, size-based metrics, and overlap-based metrics. Let A be the ground-truth volume and B the auto-segmentation volume. Two set of surface voxels of A and B are denoted by S(A) and S(B), respectively.

#### 3.1.1 Boundary-Distance-Based Metrics

We are interested in three metrics belonging to boundary-distance-based methods which relied on the definition of surface distance and quantify the dissimilarity between the surfaces of the predicted area and the ground-truth.

##### 3.1.1.1 The Average Symmetric Surface Distance

It is then given by (5), in which the value 0 represents for a perfect segmentation.


(5) $$\text{ASSD}=\frac{\Sigma_{S_{A}\in S(A)}d(s_{A},S(B))+\Sigma_{S_{B}\in S(B)}d(s_{B},S(A))}{\left|S(A)\right|+\left|S(B)\right|}$$

where *d*(*S_A_*, *S*(*B*)) is the shortest distance of an arbitrary voxel *S_A_* to *S(B)*.

##### 3.1.1.2 The Maximum Symmetric Surface Distance

The maximum symmetric Surface Distance (MSSD) is also known as the symmetric Hausdorff distance. MSSD is based on maximum distance of an arbitrary voxel *S_A_* to *S(B)* instead of the average distance like average symmetric surface distance (ASSD) as in (5).


(6) $$\text{MSSD}=\max\left(\underset{S_{A}\in S(A)}{\max}d(S_{A},S(B)),\underset{S_{B}\in S(B)}{\max}d(S_{B},S(A))\right)$$

Following (6), the output is the true maximum error. Hence, it is sensitive to outliers. However, this value is required for applications such as surgical planning, where the worst case error is more important than average errors (27).

##### 3.1.1.3 Root Mean Squared Deviation

As ASSD and MSSD, the root mean squared deviation (RMSD) is based on surface distance, which is 0 for a perfect segmentation as in (7). They are given in millimeters. However, the RMSD is highly correlated with the average distance but has the advantage that large deviations from the true contour are punished stronger.


(7) $$\text{RMSD}=\sqrt{\frac{1}{N}\Sigma_{\sigma_{i=1}}^{N}\sigma_{i}^{2}}$$

#### 3.1.2 Size-Based Metrics

Size-based metrics found the difference in size between the segmentation and the ground-truth. The best achievable results can be obtained even when the segmentation and the ground-truth are disjoint.

##### 3.1.2.1 The Relative Volume Difference

The relative volume difference (RVD), which is an asymmetric measure, calculates the absolute size differences of the regions, as a fraction of the size of the reference (8).


(8) $$\text{RVD}=\frac{\left|B-A\right|}{\left|A\right|}$$

RVD helps to recognize the method that tends to be over or under segmentations. A value of 0 for the RVD means both volumes are identical. In addition, RVD is also used to directly evaluate the volume metric information which is the single most important number that provides for applications such as liver surgery planning.

#### 3.1.3 Overlap-Based Metrics

The family of overlap-based methods is not concerned with the spatial distribution of voxels or the absolute size of the areas involved but only for the number of correctly classified or misclassified voxels.

##### 3.1.3.1 Volumetric Overlap Error

The volumetric overlap error (VOE), which is the complement of the Jaccard index, computes the ratio between intersection and union of the ground-truth A and prediction B:


(9) $$\text{VOE}=1-\frac{\left|A\cap B\right|}{\left|A\cup B\right|}$$

The value of this measure ranges from 0 to 100 where 0 for perfect segmentation and 100 for none-overlapping at all.

##### 3.1.3.2 Dice Score

The dice similarity coefficient is measured for each detected region of interest, as in (10).


(10) $$\text{DSC}=\frac{2\left|A\cap B\right|}{\left|A\right|+\left|B\right|}$$

### 3.2 Running Configuration

#### 3.2.1 Datasets

We conducted experiments on two datasets from 3Dircadb and Liver Tumor Segmentation (LiTS) dataset. For the 3Dircadb dataset, there are a total of 22 patients corresponding with 22 volumes of images. For the List dataset, 201 volumes are getting from the Liver Tumor Segmentation Challenges. The ground-truths of two datasets were provided. The 3Dircadb dataset is a subset of the LiTS dataset with case numbers from 27 to 48. Therefore, using the LiST dataset as the training data and validation on the 3Dircadb dataset is not allowed. We trained our model with 109 cases from the LiST dataset after removing the data from the 3Dircadb dataset and evaluated the performance on the 3Dircadb dataset and 70 remaining cases of LiST dataset. Data preprocessing was performed as described in *Preprocessing*. Before evaluating the primary performance of our network, we have randomly divided all images of a total of 109 cases into 80% for training and 20% for validation to determine the hyperparameters and avoid overfitting.

#### 3.2.2 Model

The proposed MFU-net was compared with the original U-net, Attention Unet model. The network settings are presented in **Figure 1**. Besides that, we used Adagrad optimizer with a learning rate of 10^-3^. All the networks were performed until 50 epochs for convergence with batch size 16. For each run, the best weight what achieved the best dice score on the validation dataset were use to evaluate the the performance of these models on the test dataset.

The evaluation measurements were introduced in section *Evaluation Metrics*. The values of RVD, ASSD, MSSD, RMSD, and VOE are the lower, the more significant. In contrast, higher dice scores are better.

### 3.3 Data Preprocessing

**Figure 3:**
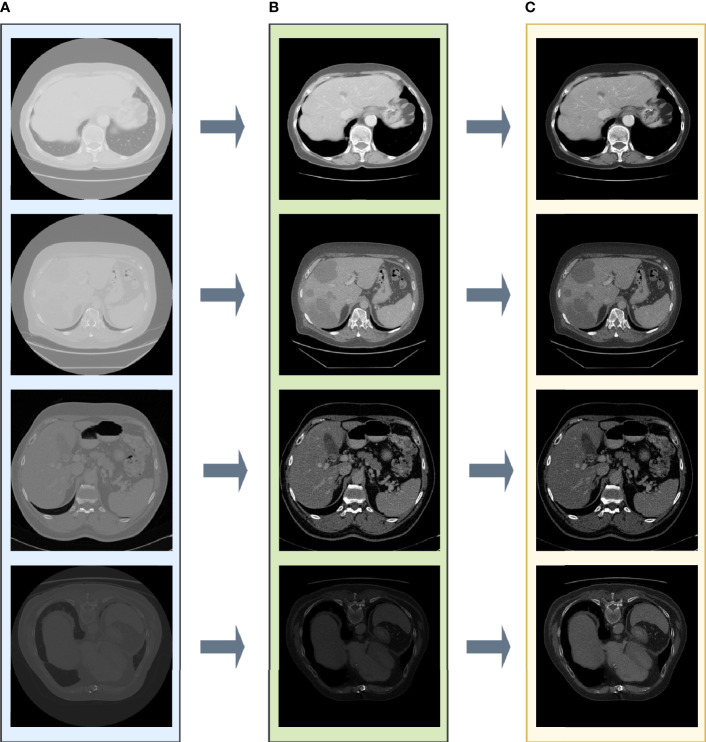
The results after each step in liver tumor preprocessing process. **(A)** The original slide; **(B)** the post-Hounsfield-filtered slide; **(C)** the poststandardized slide.

In **Figure 2**, the pixel intensity differs among different slices as well as various patients. After enhancing the contrast and gamma based on the mean value of each image, organs appear more explicit and more homogeneous (**Figure 3C**) than the original slides (**Figure 3A**) and the post-Hounsfield-filtered slides (**Figure 3B**). These outputs prove that the mean value affects the contrast and gamma enhancement in each CT slice image.

### 3.4 Performance Evaluation

In this study, we developed image processing and tumor region recognition algorithms for CT images of liver HCC. The algorithms were successfully used to visualize the liver and tumor regions on CT images in an end-to-end manner. The volume outcome is the combination of individual slices in the correct order and coordinates and has the same dimensions and the same voxel spacing as the input volume. Then the correlation between predicted volume and ground-truth volume was reported in terms of six metrics as depicted in *Evaluation Metrics*.

**Figure 4:**
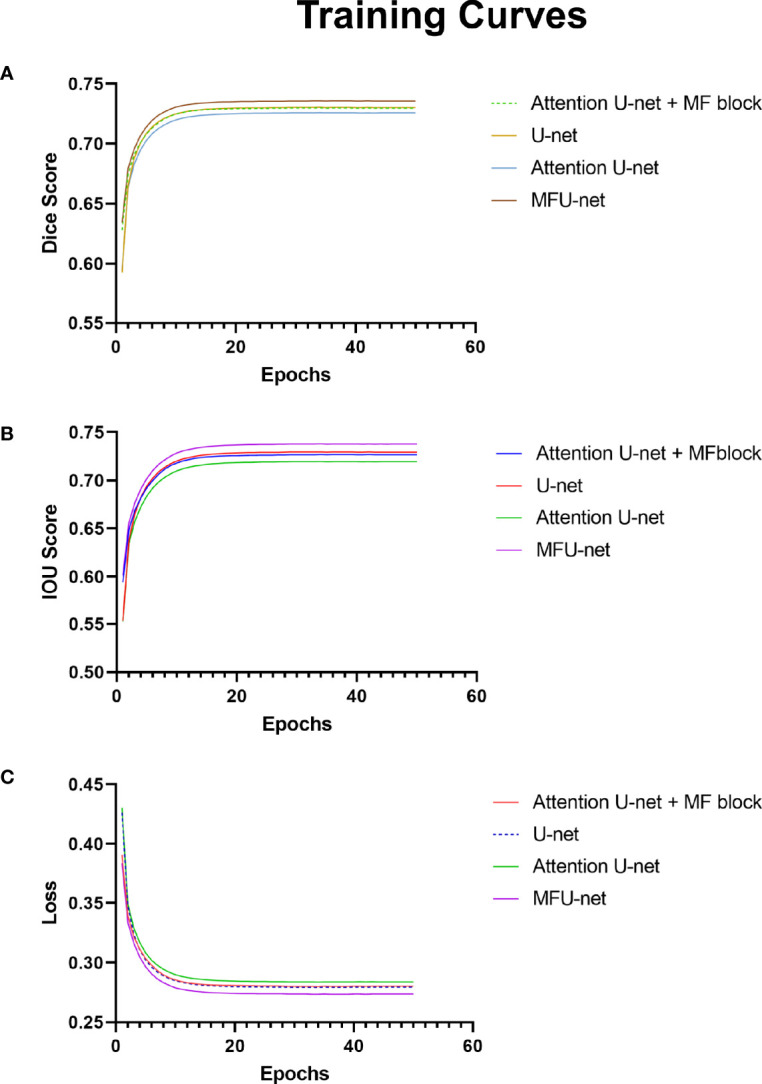
Experiments on 3Dircard training set. **(A)** Training curves of dice score; **(B)** training curves of IOU score; **(C)** training curves of loss.

These charts in **Figure 4** depict data about the training curves of 3Dircard training set to three values: dice score, IOU score, and loss. Overall, as can be seen from the graph, the MFU-net learns better than other models. The dice score and IOU score of MFU-net model were higher than those of other models by more than 1.15% on each epoch (**Figure 4A, B**) through training process. Besides that, the loss value was always the lowest in four compared models (**Figure 4C**). In addition, integrating the MF block to traditional U-net and attention U-net improves the dice score and IOU score, and at the same time reduces the loss values throughout all the 50 epochs.

**Figure 5:**
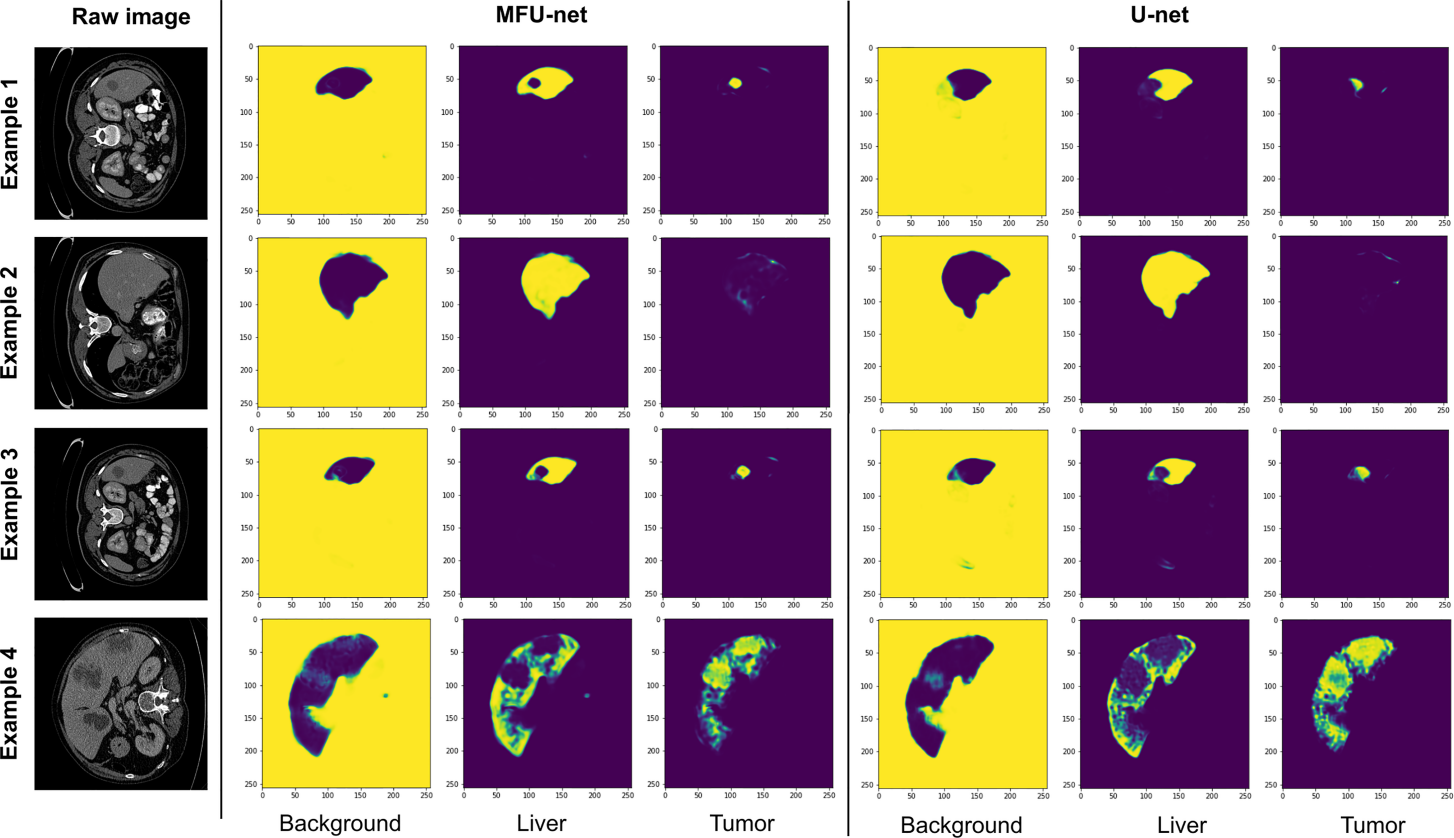
The feature maps of the last layer extracted from MFU-net and U-net comparing with raw images.

**Figure 5** depicts the examples comparing the feature maps from the last layer of MFU-net and original U-net model. There are three feature maps with a size of (512, 512) corresponding to background, liver, and tumor regions. The feature map captures the results of applying the MFU-net and U-net architectures to the same input (as the raw images shown in **Figure 5**). We found that the shape of the livers and their tumor as well as the texture features of interested objects were clearly visible in the feature maps from last layer of each architecture. However, these features are getting better through the MFU-net.

**Figure 6:**
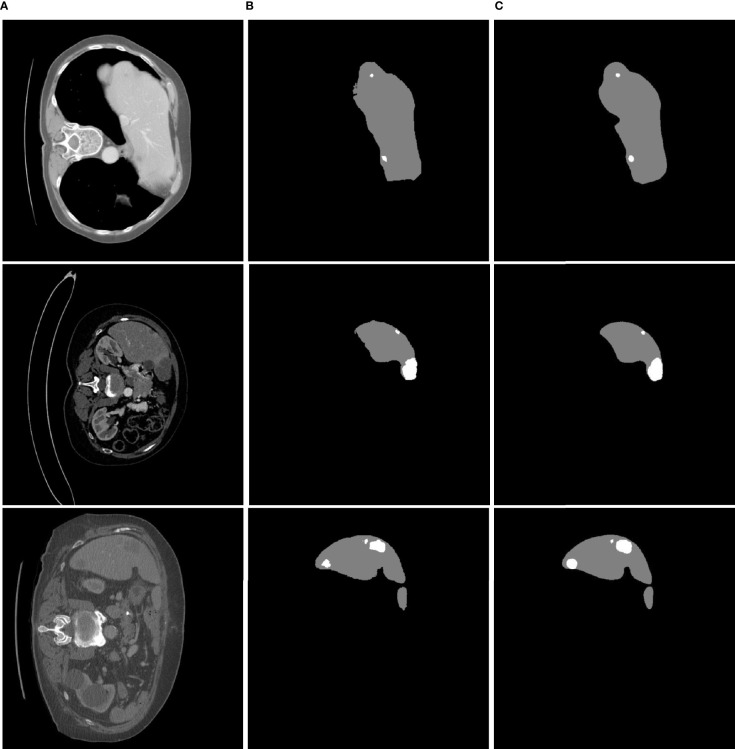
Liver tumor segmentation results using MFU-net. **(A)** Raw image; **(B)** ground truth image; **(C)** segmented tumor image.

Each class (background, liver, tumor) had a threshold of 0.5 for getting result. The liver and tumor region predicted as the category with the highest probability. In this analysis, when we used different abdominal CT images to validate the proposed approach, we found that it is less sensitive to noise during attempted extraction of liver tumors. Some example results are shown in **Figure 6**.

**Figure 7:**
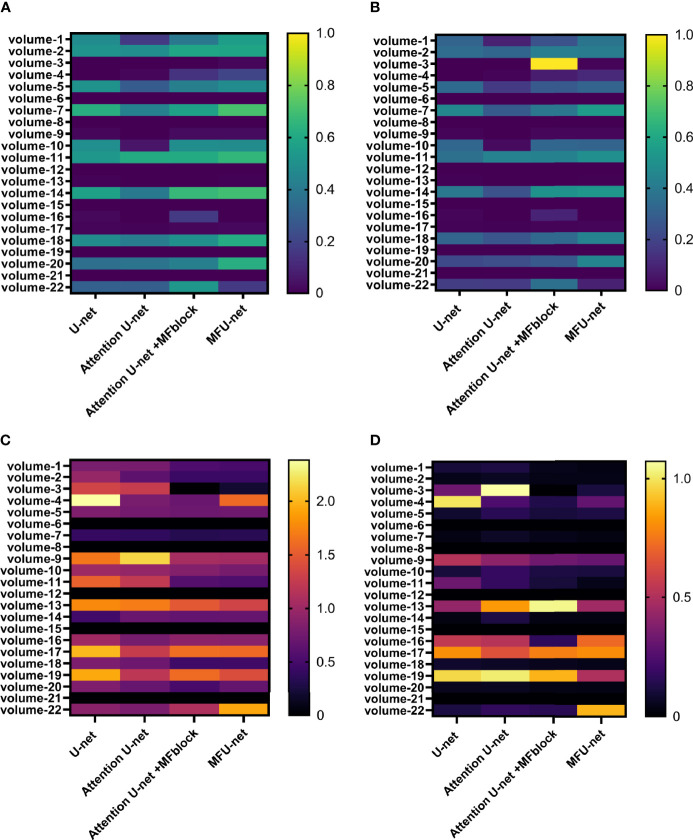
Performance on 3Dircadb dataset ***via*** four metrics: **(A)** dice score, **(B)** Jaccard score, **(C)** MSSD score, and **(D)** ASSD score.

**Figure 7** gives heatmap information about the results of the 3Dirdcard dataset with four scores: dice score (**Figure 7A**), IOU score (**Figure 7B**), MSSD score (**Figure 7C**), and ASSD score (**Figure 7D**). It is clear that while a higher dice score and IOU score is better performance, the opposite is true for an MSSD score and ASSD score. Over 22 volumes, the proposed model MFU-net predicted better results than other models. It is noticeable that the predicted score for models without MF block lagged that of models with MF block.

The performance metrics for LiTS dataset are presented in **Table 2**, including boundary-distance-based, size-based, and overlap-based metric. All compared methods was described in (28) for liver tumor segmentation results. They all achieved top rankings for at least one metric as follows: Roth et al. ranked first according to the ASSD score; Li et al. ranked first according to the MSSD score and dice score; Bi et al. ranked first according to the RMSD score; MIP_HQU team ranked first according to the RVD score; Roth et al. and MIP_HQU ranked second according to the VOE score; and Tian et al. ranked second according to the dice score. Different from their algorithms, our proposed method, the MFU-net uses an end-to-end training strategy to obtain liver and tumor all at once.

**Table 2:** Comparison between the proposed approach and other liver tumor segmentation methods on the LiTS dataset.

Methods	Boundary-distance-based			Size-based	Overlap-based	
	ASSD (mm)	MSSD (mm)	RMSD (mm)	RVD (mm)	VOE (%)	Dice score (%)
MFU-net (ours)	**0.864**	**6.035**	**1.349**	0.066	**33.50**	71.90
Roth et al. (28)	0.950	6.810	1.600	0.020	34.00	66.00
Li et al. (28)	1.073	6.055	1.562	5.164	35.60	**82.90**
Bi et al. (28)	1.006	6.742	1.520	3.431	35.60	73.50
MIP_HQU (28)	1.090	7.840	1.800	**-0.130**	34.00	65.00
Tian et al. (28)	1.189	6.682	1.726	5.921	39.40	79.40

The bold values are the best (state-of-the-art) values.

Overall, the proposed method MFU-net achieved the best results on the most of the boundary-distance-based evaluation metrics. To be more specific, our methods obtained the best ASSD score (0.864 mm, **Table 2**), MSSD score (6.035 mm, **Table 2**), and RMSD (1.349 mm, **Table 2**). In the size-based evaluation metric, our model obtained the aggressive RVD score (0.066 mm, **Table 2**). Moreover, in the overlap-based evaluation metric, the VOE score was also at the highest position (33.50%, **Table 2**), while the dice score was competing with any of the remaining methods (71.90%, **Table 2**). Consequently, our algorithm is efficient to train and effective at reducing the overlap error or distance between the ground-truth and predicted region.

Our method simultaneously recognizes the liver and tumor region. The predicted tumor performance rose over the evaluation metrics whereas the liver performance received the competitive figure with other methods (96% in dice score).

## 4 Discussion

Liver and tumor segmentation is an essential prerequisite for the effective therapy of liver disease. However, automatic liver and tumor segmentation in medical imaging remains a challenging issue. In recent years, deep learning techniques have brought the competitive performance to complex medical image analysis tasks that rely on labeled training datasets.

The proposed method is based on U-net and multiple filters to find liver and tumor regions simultaneously and accurately. Different from other existing methods, our method has two important characteristics regarding the proposed MFU-net. First, the previous liver tumor segmentation was a two-way process or cascaded approach (18, 29–33). In other words, tumor segmentation has been done after liver segmentation from the abdominal CT scan image. Here, however, liver and tumor were segmented simultaneously from the abdominal CT scan images with competitive performance. This reduces the time and effort needed during the liver tumor segmentation process. Second, the final segmentation results do not directly depend on any post-preprocessing method such as level set (34), CRF (35), object-based (36), active contour (29), and so on.

Additionally, the problems that arise in the three-dimensional imaging segmentation are the complexity of the surface and its folding as well as the ambiguity of the correct surface topology on complex voxel sets (37). The region of interest and its expected boundary can be concealed and are therefore challenging to segment. This research provides an accurate scheme to alleviate the surface distance between the ground-truth volume and autosegmentation volume by considering the effects of multiple filters compared with a single filter.

To demonstrate its capabilities, we performed experiments to compare its performance with U-Net, Attention U-net through visible illustration, quantifying the difference between architectures using four metrics on the 3Dircadb as shown in **Figure 7**. We then performed statistical tests to compare the metrics from the proposed method and other methods on the LiST dataset (**Table 2**). The results show that the proposed method has significantly improved performance than other methods on most metrics, especially boundary-distance-based metrics.

## 5 Conclusion

We introduced the method for the liver vs. liver tumor segmentation that serves as an objective, end-to-end recognition method. The MFU-net is an architecture that combines the multiple filter block based on the U-net architecture. The multiple filter block can be integrated into other deep learning networks. The results were analyzed on two datasets (3Dircadb and LiTS) by several measurements to show the improvement of our proposed method. Multiple filters efficiently learn contextual features of two dependent objects (liver and tumor), minimize the surface distance errors, and can deal with liver and tumor shape diversity. Simultaneously, they are less sensitive to contrast and gamma complexity in CT images. The liver tumor region generated by our model can help radiologists locate tumor regions on CT images swiftly and accurately. The model development pipeline can be used in other organ and tumor types. In future work, we would extend this segmentation to other common tumor types in order to aid better treatment diagnosis.

## Data Availability Statement

The LiTS dataset were obtained from the competition at https://competitions.codalab.org/competitions/17094. The 3Dircadb are available at: https://www.ircad.fr/research/3dircadb/.

## Author Contributions

All authors listed have made a substantial, direct, and intellectual contribution to the work and approved it for publication.

## Funding

This research was supported by the Bio & Medical Technology Development Program of the National Research Foundation (NRF) and funded by the Korean government (MSIT) (NRF-2019M3E5D1A02067961).

## Conflict of Interest

The authors declare that the research was conducted in the absence of any commercial or financial relationships that could be construed as a potential conflict of interest.

## Publisher’s Note

All claims expressed in this article are solely those of the authors and do not necessarily represent those of their affiliated organizations, or those of the publisher, the editors and the reviewers. Any product that may be evaluated in this article, or claim that may be made by its manufacturer, is not guaranteed or endorsed by the publisher.
